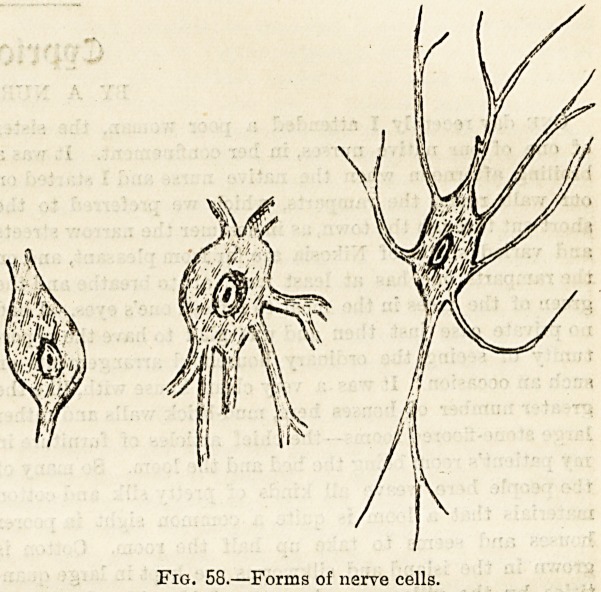# The Hospital. Nursing Section

**Published:** 1902-09-06

**Authors:** 


### The Hospital.

### Nursing Section.

Contributions for this Section of "The Hospital" should be addressed to the Editor, "The Hospital" Nursing Section, 28 & 29 Southampton Street, Strand, London, W.C.

No. 832.—Vol. XXXII. *SATURDAY, SEPTEMBER 6, 1902.*

### Notes on News from the Nursing World.

### PRINCESS HENRY OF BATTENBERG AT NETLEY.

Last week Princess Henry of Battenberg paid an unofficial visit to Netley Hospital, being accompanied by Princess Victoria of Schleswig-Holstein and Princess Hohenlohe. The visit was greatly appreciated by the patients, to whom the Princesses spoke very kindly, and to some of the more helpless they presented small bunches of flowers. Before leaving, Princess Henry expressed her approval of all she saw, and thanked Miss Norman and Surgeon-General Townsend and others of the staff who conducted them round the wards. It may be mentioned that neither on this, nor on any previous, visit of Royalty to Netley Hospital were the wards decorated with flowers and then stripped after the visitors left. This visit nearly ended in a disaster. On leaving the hospital Princess Henry preferred to walk the short distance to the pier, while Princess Victoria, Princess Hohenlohe, and Colonel Colborn drove down in an open carriage. When the pier was reached the horses took fright at the noise of their own feet on the boards and the glitter of the water, and but for the coachman's presence of mind in turning them back—in doing which the pole was broken—would probably have dashed over the pier end. They were stopped by Private Evans, one of the convalescent patients, assisted by others, while Private Tuck, of the Manchester Regiment, an eye patient, pluckily jumped in the carriage and with the aid of another man managed to get the young Princesses out before they were hurt and before the horses, which plunged about a great deal, were brought to a standstill, the dangling of the pole between their legs adding to their fright. Naturally, the Princesses were much frightened and shaken, the shock causing Princess Hohenlohe to faint soon after being taken out of the carriage; but, happily, the suspense only lasted a few minutes, and then the Royal party, after expressing their thanks to their deliverers, proceeded on their way to the launch.

### NURSES FROM SOUTH AFRICA.

The arrival of the following nurses from South Africa is announced :—On the *Gascon*, Sisters M. G. Denton, A. Matheson, S. J. Coldwell, E. Gray, E. R. Tait, and G. McFarlane (Civil); on the *Arundel Castle*, Sisters A. S. Johnston, B. J. Bettys, B. Gilbert, A. W. Britten, E. A. Boyce, E. Cockburn, B. Crocker, E. A. Hancock, and E. W. Jayne; on the *Braemar Castle*, Sisters S. Clarke, A. McLeod, M. C. Reilly, A. M. Lashwood, H. Wohlford-Hansen, E. Smith, and, as invalid, L. Cruickshank; on the *Moravia*, Sisters F. E. Ridgeley and E. F. Beedie; on the *Aurania*, Sisters F. H. Barry, K. Webb, A. B. Noble, E. M. Beetham, B. E. Caws, M. J. Mill, M. A. C. Millington, G. K. Swanton, V. P. Squire, E. Johnson, E. M. Leudon, M. M. Tinsley, M. E. Richardson, C. E. A. Thorpe, P. M. Barsdorf, L. E. Colston, E. A. Chaffey, M. Ainsworth, E. A. S. Webster, C. Kitching, A. E. Byrne, G. Deacon, F. M. Learmonth, E. Prangley, D. E. J. Wetton, E. Ellis, M. Talbot, E. McIntosh, A. A. Bousfield, H. H. Mason, and A. A. Austin (New South Wales); on the *Nubia*, Superintendent Sister C. H. Keer, Sisters A. R. Cullum, K. Despard, L. M. Green, J. Paget, M. Pedler, F. Sykes, K. Phillips (Civil), as invalids, Sisters H. M. Shaw, A. B. Denton, S. M. Patterson, and K. Finnemore (Civil); on the *Norman*, Sisters G. Hodge and L. Dawson; on the *Norham Castle*, Sisters E. Selighmann and C. Strahn. It is interesting to learn that of the 31 nurses who came home on the *Aurania* none had applied for leave, but were all sent home on reduction of the establishment.

### A BISHOP ON QUEEN'S NURSES.

At a meeting held in the Bishop's Palace at Hereford in support of the Women's Memorial to Queen Victoria, the Bishop of Hereford said it was important to bear in mind that the main objects aimed at by Queen Victoria's Jubilee Institute were not only to nurse the poor in their own homes, but to help, and often to instruct and educate the people as to the keeping of their homes healthy and bright, and thus improving all the conditions of home life. "He thought that they were all agreed that one of their greatest needs in both country and town life among the poor was to have more of that kind of skilled assistance which the trained nurse brought into the home in time of sickness." Dr. Percival expressed the hope that in a county like Hereford it might be possible to group parishes together so as to make a district sufficiently large to support a Queen's nurse, "and also, perhaps, employ one or two assistants of a somewhat cheaper character." As to the last point, Lady Elizabeth Biddulph stated that the management committee contemplated the subsidisation of nurses sent out to poor parishes, which would thus be able to obtain them at a lower rate.

### THE SCARCITY OF JEWISH NURSES.

A Jewish contemporary avers that "the scarcity of Jewish nurses has long been one of the inexplicable mysteries of our communal life," and recalls the fact that at the Jewish Women's Congress in May it was declared that no Jewish nurse could be found for the children's block of the Baroness de Hirsch Convalescent Home, which, it says, is "not very creditable when one considers the growing numbers in which cultured Christian women embrace one of the most beautiful and sacred of all vocations." We, of course, believe that a woman's capacity to nurse does not depend upon whether she belongs to the Anglican or Roman Church, the Jewish persuasion, or one of the great Nonconformist denominations. But we can quite understand the desire of pious and philanthropic Jews that there should be Jewish nurses attached to some of the leading hospitals; and that there is, in particular, scope for the labours of midwives of Jewish birth and sympathy in the East End of London, is proved by the evidence recently given before the Alien Commission. The heads of the nursing profession would, we are sure, rejoice if a larger proportion of Jewish young women, fired by a desire to tend the sick, and imbued with the true nursing spirit, sought admission at training schools.

### THE HOME SISTER'S AUTHORITY.

Last week a Liverpool nurse wanted to know, in substance, whether the home sister of a hospital has any authority over members of the staff when they are off duty and not wearing uniform. A very fair answer is, we think, given to her in our issue of to-day by another correspondent, who reminds her that when nurses sign an agreement to work for a hospital they are under the control of their official superiors, both on and off duty, until their period of training has expired. We also entirely agree with her remark that "training" does not only mean nursing but includes conduct and a sense of the fitness of things. A home sister, whose responsibilities are almost invariably considerable, cannot fail, if she take any real interest in the nurses under her, to observe their dress and demeanour generally, though, of course, she should be careful in the exercise of her authority to temper it with tact.

### DUBLIN NURSES AND DROGHEDA GUARDIANS.

Apparently the Dublin nurses have made up their minds to fight shy of the Workhouse Fever Hospital at Drogheda. Dr. Adrien told the Drogheda Board of Guardians at their last meeting that, in consequence of the prevalence of typhoid fever in the Workhouse Hospital, he had wired to the Dublin hospitals for a nurse, but could not get one, "the reason being that when nurses came from Dublin to Drogheda on former occasions they were not well treated." The chairman, who said he had not heard of any complaints of ill-treatment before, asked for details, and it subsequently transpired that there had been friction between the then master and the nurses—a by no means unusual event. Dr. Adrien also stated that meat, egg-cups, and "other things too numerous to mention," which the nurses needed, had been refused to them. Eventually, the present master was requested to proceed to Dublin in search of a nurse. It may be hoped that he is an official who recognises that nurses cannot reasonably be expected to accept service in an institution in which there is a difficulty in obtaining the necessaries of life.

### THE ALNWICK NURSING ASSOCIATION.

In connection with our remarks under the heading of "A Collection Refused," in The Hospital of August 23, it is due to the Duke and Duchess of Northumberland to mention that the Nurses' Home at Alnwick has just undergone a thorough repair and renovation at their expense. To their liberal support it is also, we understand, largely due that the Alnwick District Nursing Association had in hand at the end of the financial year the substantial balance of £195 13s. 7d. This is very satisfactory, but we nevertheless think that it was a mistake on the part of the Duke and Duchess to refuse the proceeds of a collection at a church parade of cyclists.

### UNSCRUPULOUS HOME KEEPERS.

There is no doubt that one of the causes of the prejudice which is entertained in some quarters towards private nurses is the existence of unscrupulous keepers of nursing homes. These people do not hesitate to take into their employ women who are unable to give credentials of character. An instance of the kind is before us. The matron of a home in a London suburb states that she had occasion, a little time ago, to summarily dismiss a nurse in her employ for insubordination, refusing duty, and unkindness to, and neglect of, a helpless patient. This nurse, the matron learnt, had since been engaged on the staff of another institution in the far west-end of the metropolis, without any inquiry as to her antecedents on the part of the proprietor. She accordingly called upon him and told him how and why the nurse was dismissed, and he said that he would not keep her. But she has ascertained that the woman is retained as one of the staff. It is obvious that so long as there are proprietors of homes, whether men or women—though doctors and the public would do well to avoid nursing organisations whose staff consists of women and is run by men without a qualified matron in charge—who will engage nurses without full investigation as to both their training and subsequent work, the profession must continue to be injured by including in its ranks individuals who are a source of discredit.

### CAUSE AND EFFECT AT BARROW.

The Barrow Board of Guardians might not have been much concerned at the resignation of an assistant nurse because she did not have sufficient time for recreative purposes, but when the superintendent nurse resigned for the same reason they were evidently impressed, and at their last meeting they decided that the nurses should have 11 hours in the week free to themselves. The concession would have been more graceful if it had not been secured by force of circumstances.

### THE NEW HOME AT PRESTON.

On Tuesday the Countess of Derby formally opened the nurses' home which, under the title of the Diamond Jubilee wing, has been built as part of an extension of the Preston and County of Lancaster Queen Victoria Royal Infirmary. The home, which, has been in use for a few months, provides accommodation for about forty nurses, and obviates the necessity of "living out," a bad practice which can only be defended when circumstances render it unavoidable. The Robert Charles Brown operating-theatre was opened at the same time, and the numerous guests inspected the extension. The nurses are greatly pleased with their new home.

### THE CREDITON BAZAAR.

As the result of the recent bazaar in aid of the Crediton District Nursing Association, at Downes, the bazaar committee have handed a cheque for £141 4s. 7d. to the president, Lady Audrey Buller, who expressed her thanks to the committee and stall-holders.

### Lectures to Nurses on Anatomy.

By W. Johnson Smith, F.R.C.S., Principal Medical Officer, Seamen's Hospital, Greenwich.

### LECTURE XXV.—THE ORGANS OF THE NERVOUS SYSTEM.

By the proper and natural working of the important parts constituting the nervous system, the combined mass of all other organs and structures described in these lectures is rendered, so long as vitality persists, a sentient, active, and self-regulating organism.

These important parts—the organs of innervation—are arranged into two systems, (1) the *cerebro-spinal system* and (2) the *sympathetic system.*

The *cerebro-spinal* system consists of two central organs which, as they are continuous, form what is called the *cerebro-spinal axis*; the *brain or encephalon* enclosed within the skull, and the *spinal cord or marrow* occupying more or less of the spinal canal according to age; and a great number of ramifying and interlacing cords known as *nerves* attached by their central ends to the brain or cord and connecting one or both of these central organs with different parts of the body, especially with muscles, skin, and the organs of special sense.

The *sympathetic system* which acts for the most part on blood-vessels and internal organs consists of (1) an extensive and widely diffused set of minute nerve centres—brains in miniature—which are called *nerve ganglia*; and (2) minute nerves like threads, some of which connect sets of ganglia, whilst others are given off by the ganglia in two directions, on the one hand to blood-vessels and the organs supplied by these vessels, and on the other hand to the cord and to the nerves of the cerebro-spinal system.

The sympathetic ganglia are arranged in two long chains extending on either side of the spine from the base of the skull to the coccyx—3 ganglia in the neck, 12 along the thoracic portion of the column, 4 along the lumbar portion, and 5 in front of the sacrum. They are also collected in groups in each of the three large cavities of the body—the head, chest, and abdomen—forming with the connecting nerve fibres distinct networks called *plexuses*, from which are directly sent off, and through which are transmitted from the cerebro-spinal system, innumerable thread-like nerves to the arteries which carry blood to the brain, the heart and lungs, the gastro-intestinal canal, and other internal organs.

It should be remembered that the cerebro-spinal and sympathetic systems, although they differ with regard to their respective functions, their anatomical arrangement, and to some extent in their minute structure, are closely associated in their distribution throughout the body, and in their working are mutually dependent.

An anatomical section of a fresh brain or spinal cord shows that these centres are not composed of a simple and uniform material. The soft white tissue commonly regarded as a characteristic feature of nerve structure will on the surface of such sections be found associated with a tissue of a distinct grey or reddish grey colour, which is arranged either as a continuous layer or a continuous column, or in scattered deposits or islets. This difference in tint indicates very important differences in both structure and function, the *grey*, or as it is sometimes called the *cineritious matter*, being composed of cells or corpuscles and constituting the active element of innervation or special nerve function, whilst the *white matter*, which is made up of long threads or fibres, serves mainly as a transmitting or conducting agent. The grey material may thus be compared to the battery or generating centre of an electrical machine and the white matter to the conducting wires.

The *grey matter* is present (*a*) on the surface of the brain where it forms a thin continuous layer called the *cortex or cortical portion*; (*b*) in the interior of the brain where it is concentrated in certain distinct centres and thin sheets; (*c*) in the interior of the spinal cord forming along its whole length a central axis with anterior and posterior extensions known as *cornua* or horns; (*d*) in the ganglia of the sympathetic system and in ganglia attached to the roots or origins of nerves of the cerebro-spinal system. The cells or corpuscles contained in this grey matter vary much in size (300th to 4,000th of an inch) and also in shape. They are characterised chiefly by their tendency to send off limbs or prolongations (fig 58) which in extreme forms, such as

Fig. 58.—Forms of nerve cells.

exist in the grey matter of the spinal cord, are very long and numerous, and are often broken up into intricate arrangements of branches and twigs.

The nerve-fibres composing the *white matter* of the brain and spinal cord are long and very minute threads (from $\frac{1}{3000}$th to $\frac{1}{6000}$th of an inch in diameter) which are continuous with the prolongations from the nerve-cells. Arranged in bundles which are bound together by an organic cement of delicate fibrous tissue, the nerve-fibres constitute the nerves which pass from the two central organs of the cerebro-spinal system, and are widely distributed just as blood-vessels are through the different parts of the body. The nerves derived from the brain and cord when examined under the microscope soon after death are found to be dark-bordered, each presenting a central rod of soft and translucent nerve-tissue and a distinct sheath of fatty matter. These fibres are called *medullated or white* fibres, and are thus distinguished from the smaller and more delicate fibres of the nerves of the sympathetic system which, as they do not possess a sheath and are darker in tint, are called non-medullated or grey fibres.

The pale or sheathed nerve fibres so widely distributed throughout the body, though they vary but slightly in structure, claim with regard to their mode of working an important two-fold division. Each nerve fibre, so long as it is sound and intact throughout, acts like a telegraph wire, as a conductor transmitting messages, or as physiologists say, nervous impulses or currents between the nerve centres and the other organs of the body. Though each of these conducting fibres always conducts in the same direction, the direction differs in each of the two main groups in which

### LECTURES TO NURSES ON ANATOMY.—*Continued.*

nerve fibres are arranged. In one group, that of *efferent or motor fibres*, the direction of the current is *from* the brain and *towards* other parts of the body, these fibres bearing the mandate of the will to different muscles, and effecting what are known as voluntary movements of the limbs and trunk. In the fibres of the other division, as their titles of *afferent or sensory* nerve fibres imply, the current passes in the reverse direction, and *towards* the brain and cord *from* the organs of touch and of special sensation at or near the surface of the body. In most of the large nerves of the trunk and limbs the two varieties of nerve fibres, the motor and the sensory are mixed together, but near the nerve centres—the brain and cord—they are separated and arranged into two sets of nerve roots or trunks, these roots being composed exclusively of motor fibres in one set, of sensory fibres in the other set. Section, crushing, or destructive disease of either of these roots causes more or less complete nervous isolation of the parts to which the nerve fibres in the affected root are ultimately distributed. Thus, if the motor root be divided or seriously injured, the muscles in relation with this root will become quite useless or paralysed, and if, on the other hand, it be the sensory root alone that is thus impaired, there will be no failure of muscular power but a loss of sensation in that part of the surface of the body, whether a region of skin or an organ of special sense, to which its constituent nerve fibres are distributed.

### Cypriot Babies.

BY A NURSE IN CYPRUS.

One day recently I attended a poor woman, the sister of one of our native nurses, in her confinement. It was a broiling afternoon when the native nurse and I started on our walk round the ramparts, which we preferred to the short cut through the town, as in summer the narrow streets and varied smells of Nikosia are far from pleasant, and on the ramparts one has at least fresh air to breathe and the green of the trees in the moat to refresh one's eyes. I had no private case just then and was glad to have the opportunity of seeing the ordinary household arrangements on such an occasion. It was a very clean house with, like the greater number of houses here, mud-brick walls and rather large stone-floored rooms—the chief articles of furniture in my patient's room being the bed and the loom. So many of the people here weave all kinds of pretty silk and cotton materials that a loom is quite a common sight in poorer houses and seems to take up half the room. Cotton is grown in the island and silkworms are kept in large quantities by the villagers. A cotton-field with the brown pods just opening, like tiny cricket balls bursting at the seams, and the fluffy contents puffing out, is such a pretty sight, and the silk bazaar on Fridays, when the women come in from the different districts and sit behind their low tables, on which are spread the pretty silks and crepons, in most cases evolved from the raw materials by their own labour, is well worth seeing. The silks are very narrow, but such lovely shades of lilac, pink, yellow, or green, besides more sober colours—and the white silk and cotton crepons are perfectly charming. One feels quite hard-hearted in bargaining with a woman for her goods, knowing that she has probably fed the silkworm patiently for weeks, and spun the silk herself, paid for its dyeing, and then woven it by hand on her loom. Still here, as in most eastern countries, one must bargain, and even then, as a rule, be woefully imposed upon.

### The Patient's Bedroom.

But I have wandered far from my room and must come back to the bed, which was a large, iron four-poster, to allow of being hung with mosquito netting, and, as usual, much ornamented with crochet work on valance, sheets, quilts, and pillow-cases—the pillows are always stuffed very hard with wool, and often covered with red or blue under the white covers to show off the crochet—they must be very uncomfortable to sleep upon. They do not need any smoothing or shaking, for the people evidently prefer elegance to ease, and like them as they are. This particular bed was very clean and well supplied with old folded sheets. Nurse had also found a big zinc bath for baby's first tubbing, which, from its size, must have been the family wash-tub; but there seemed no clothes provided for the tiny newcomer, except a number of little long-sleeved cotton coats and caps and pieces of calico, new and old. The room seemed full of people when I arrived, but they all turned out into the passage very quietly, and contented themselves with taking occasional peeps at what was going on through an opening in the wall, which they had to stand on a chair to reach. Nurse and the old grandmother (a little, shrivelled-up, brown woman, with such bright eyes, who looked about ninety) stayed in the room, and everything was soon over without any trouble, for it was a normal labour, and I had brought all I needed with me. The only difficulty was in getting plenty of hot water, for they have only earthen pots of charcoal as fires, and generally one big cooking pot which is used for every purpose and has to be dipped into for small quantities of water.

### The Amusing Part of the Performance.

When the mother was comfortably settled, I turned my attention to the baby, and then the amusing part of the performance began. The bath was so large that all the water had to be tilted into one end for poor baby to get any benefit from it. I had made them find a strip of flannel for the binder, but when that was sewn on and one little coat and piece of calico adapted to circumstances I was at a standstill, and nurse finished the toilet "à la Cypriot," whilst I looked on. Poor baby was laid flat with its arms at its sides and carefully and tightly rolled in one piece of stuff after another till it was a complete little mummy; then a frilled cap was tied on so that nothing but its little red face was visible, and it looked so comical. I persuaded them to leave its arms out next day, but baby seemed quite happy in chrysalis form. In the ordinary course of things she would not have been bathed for a day or two, but would have been well rubbed with a mixture of wine and salt, which would have been left on to soak in and strengthen the child; the eyes would have been bathed with wine and then daubed round with a mixture of oil and charcoal. The swaddling clothes are used for about two months. These customs explain the Bible references to "salting" and swaddling which sound so strange to the uninitiated. When baby's bath does take place at last, the friends all come to the ceremony, which is performed by the midwife, and throw coins into the bath, which become her perquisites. She is also usually loaded with cakes and sweetmeats. Baby's head is, as a rule, left strictly alone, the top of it being considered such a tender part that a thick cake of dirt and matted hair is supposed to be a great protection. We have had cases in hospital where the cradle-cap needed a poultice to remove it, and the mother quite felt in each case

### CYPRIOT BABIES—*Continued.*

that the child had been deprived of a long-treasured safeguard to its tender skull.

### A Paradise for Mrs. Gamp.

The next stage of the performance was refreshments. Mrs. Gamp would have been quite happy in a Cypriot house in that respect, for people are very hurt if you don't partake of their special delicacies when you visit them, and she might have had a "bottle on the mankelpiece" very inexpensively out here, for both native wine and brandy are very cheap, and I have known a festive party of about nine people quite incapable next day, after indulging in wine to the amount of 1s. 6d. between them. "Sairy" might not have found the mantelpiece certainly, but there would have been no difficulty over the spirit. Coffee and sweetmeats, however, are the usual things offered to guests, so a tray soon came in with a glass of preserved apricots, a glass of water and spoon. The custom is to take a spoonful of preserve and then a drink of water, then a tiny cup of coffee without milk. In richer houses the coffee cups are often of very delicate china without handles and fit into silver filagree cups like egg-cups, so as not to burn one's fingers. Cigarettes are also offered, and when there are many guests the sweetmeat tray is provided with a glass of water and spoon for each person. In the chief Cadi's harem, which I once visited, here the trays and cups were richly embossed, and were handed round by black slaves who make very picturesque waitresses, they are so graceful.

### Exorcising the Evil Spirit.

Nurse was allowed to stay with her sister for a few days and came back leaving her very well, but to my surprise I heard that she had taken a walk upstairs on the second day after her confinement. There had been a heavy storm and the rain had come into their downstair room compelling her to move upstairs, but I couldn't understand why she had been allowed to walk for she was a little thin woman, and her husband a strong man who could easily have carried her. I have heard since that they usually get up on the third day, and that until that time they must never be left alone, some one, if only a child, must be in the room. The idea seems to be that the evil spirit may change the child, something after the style of the old superstition of fairy-changelings. On the third day they mother walks round the room and makes a cross in each corner. I suppose after this she is considered able to deal with the spirits single-handed, for no further companionship is needed. However, nothing seems to hurt some people, and this particular woman is flourishing now and baby is growing a big strong girl, and came in for a pretty pink hood off our Christmas tree, much to her mother's delight. I have not seen a baby christened yet and am rather looking forward to the event, for I hear that they are always undressed and completely dipped, and generally yell lustily. No wonder, poor little mites. Fancy being obliged to take a bath in public like that! But it is warm water and generally warm weather too.

### Greek and Turkish Midwives.

Midwifery, as might be expected, is not in a very advanced stage of progress in Cyprus, although there are numbers of both Greek and Turkish midwives at work in the island. Some of the Turkish ones are, I believe, fairly trained at Constantinople, as it is so usual for the Turkish women not to permit a doctor to attend them—though many of them here are glad to have our chief medical officer, who speaks their language well, and many come quite willingly to the hospital for treatment and operation. We do not take maternity cases. Sad tales of the ignorance of village midwives are continually coming to one's knowledge—such as in a cross-presentation, putting the poor woman in a blanket, held by several people, in which she is violently shaken up, to correct the presentation and turn it into a normal one.

### Classes at the Hospital.

Classes for the midwives have been held in connection with the Government Hospital, and a fair number of women attended and seemed interested, but there are many difficulties in the way. It is hard to make things plain enough to such ignorant people without being able to show them practically how the mothers and babies should be treated both at the confinement and afterwards. A small hospital, at which they could be trained, two or three at a time, would be the best plan, for district work would be impossible for English nurses to undertake during six months of the year. The midwives could be drafted back to their villages after training. Of course this means money, and Cyprus is poor; but it may be done some day.

### All He Had.

A busy Yorkshire manufacturing town, with grimy air, and grimy faces, but true, kind hearts beating everywhere. On a breezy hill, away from the noise of the town, stands the great Infirmary—the pride of the people, and with good reason. One man, breathing his last minutes of life away, this glorious June afternoon, wonders dreamily for a moment, if this can be Paradise. Are those white-capped, smiling nurses angels? And there is music too out in the distance, where earth is ablaze with Eden loveliness. Then some old words—"No more pain" come to his mind, and he knows it is all a mistake. His brain is clearer than usual, somehow. He had always been known as "Silly Bill," and had perhaps earned his name. Everybody in his little village knew the tall, thin figure, with bent shoulders and shuffling walk, the wistful brown eyes staring into vacancy. But it was only for the very few to see the far-away light behind that vacant stare—a glimpse of the numbed soul which had been alive once and would yet live again. And to-day, when he was enjoying an outing in the big town, the sleeping soul had been roused by the sight of a little child in danger from a runaway horse. Bill had not hesitated to risk his life, and the little one was given back to its mother, safe and happy. But Bill, seriously injured, was carried off to the Infirmary, and the doctors gave no hope.

"We shouldn't ha' thought it of him," said some of his neighbours who had never seen the far-away light. "Whatever made him do it?"

What? Bill is just telling the sweet-faced nurse. "I doan't get to church reg'lar, . . . but t'other Sunday I went . . . and t' parson were talking about giving . . . and I couldn't think what a chap like me . . . had to give. I've no brass (money), and folks say I've so little wit, 'at I doan't know I'm baat (about). They're wrong i' that, I do know. But when I saw t' little 'un i' t front o' t horse, it came to me 'at I could give myself. 'Twas all I had, nurse and I gave it . . . I'm glad to ha' done it . . . and I want nowt (nothing) for it. But nurse . . . my life's gone . . . and nowt to show! I'm feared 'at I shall . . . be shamed . . . i' t Great Day! P'raps . . . do you think this . . . 'll count aught for 'Silly Bill'?"

And the sweet-faced nurse, her eyes brimming over with tears, whispered softly—"Yes."

### Nursing Private Patients in Egypt.

BY AN OCCASIONAL CORRESPONDENT.

To most nurses at one time or another during their career comes the opportunity or necessity of going abroad. Usually the summons allows scant time for preparation, yet if the nurse is to arrive fit and well, ready for the duties awaiting her, it is but right that she should endeavour to travel with as much comfort as circumstances permit.

A knowledge of French or German is of course a great help abroad, and this many nurses possess, but more, like myself, have only a smattering, scanty relics of school-days. To these an account of a recent trip to Egypt may be of service. At anyrate it may help them to avoid the mistakes I made. It being necessary that I should reach my destination as quickly as possible, the sea route was out of the question, and overland to Marseilles and thence by Messageries Maritimes boat to Alexandria was, we decided, the best way.

On my arrival at Marseilles, worn out, crumpled, dirty, and dejected, I tumbled out into the fresh morning air wondering half stupidly what I ought to do next. I did not deliberate long—a bed and a bath I must have. It only remained how to obtain them.

An official with M.M. on his cap speedily solved the difficulty. Taking my baggage check and keys, he informed me I would find all in my cabin, and as the steamer did not sail till four o'clock, he took me to a neighbouring hotel, where, after drinking a cup of coffee and ordering some tea to be brought to me at two o'clock, I speedily sought my room and much needed rest. For this accommodation and the above-mentioned refreshment the bill amounted to twelve francs, a sum which would have been much more wisely invested in a night's comfortable rest in Paris.

It seemed to me I had barely closed my eyes when I felt myself being vigorously shaken, and woke to find an excited garçon alternately pointing to the table on which stood my tea and to his watch, the hands of which to my dismay stood at half-past three. What a scramble I had to be sure, arriving, however, in ample time to catch the boat.

### The Voyage.

Judging from former experiences on English and American lines I had imagined that once on board my difficulties would be at an end, and I should soon find myself among friends, for nowhere is the average Anglo-Saxon more at home than on board ship, nowhere does he show himself so accommodating or so friendly disposed. I experienced another disappointment. The rush to warmer climes as winter approaches is nowadays so great that steamers all carry even more than their complement of passengers. This particular one was crowded, but she carried but nine fellow-country folk all told, and as these, with the exception of two maids, were all travelling first-class, I found myself as lonely and as far off having anyone to speak to as ever. The crowd represented all nations, and peoples, and tongues, and every shade of colour from the ebon visaged Soudanese gentleman whom I found seated on my right at table through the innumerable browns and olives of Japs, Greeks, Syrians, and Italians, to the blue-eyed, flaxen-haired Swedes who sat opposite. The majority, however, were French. Now we English, in spite of our boast of ruling the sea, are by no means exempt from the ills of sea-sickness; but after travelling in a French boat filled mainly with French passengers, one understands why the French lost Trafalgar.

Leaving Marseilles with its wide tree-shaded promenades, bright cafés, and gay crowds, we sailed out into a rose and violet sea. Past the grim Chateau d' If of Monte Cristo, past the long, not unpicturesque buildings of the Quarantine Station, out into the rosy glow, till looking back one saw in beautiful panorama the white glittering town set in green, the vine-clad slopes and chapel-crowned heights.

Three French women shared my cabin. They chattered on for hours, but they were kind in their way, and we kept having short and halting conversation. Though as the days passed and I had to use French, it was surprising how much I had forgotten came back and how much I could understand of the general conversation. There was none of the jollity and fun, the games and gaiety with which—after the first day or two—one usually associates a sea voyage, and yet this particular voyage was one of the loveliest and most interesting, and in spite of a spice of loneliness was to me one long delight.

But even the beauty and glamour could not make one forget the mundane matter of afternoon tea, and alas there was no tea. Meals consisted of café au lait at 8 A.M., tepid in temperature. Dejeuner at 10.30. Starting with shrimps and radishes one went through such courses as harricots, lobster, stewed beef and carrots, eggs—nearly raw—fish, chipped potatoes, mutton—warmed through—and fruit, washed down with a little wine and much water, and ending with black coffee. Dinner, at 6.30, consisted of much the same dishes with a few extra courses. At 9 P.M. tea was served—a lemon-coloured fluid flavoured with condensed milk. Never again do I go on a French boat, or abroad at all, without spirit lamp and kettle, tea, sugar, Bovril, biscuits and milk, for, oh, how hungry I used to get between three and six o'clock.

As we travelled on each night the skies changed to a deeper, more velvety violet, the stars became more golden, twilights shorter. The cabins got hotter, more airless, sleep more impossible, until the grey dawn brought a rush of cold air at whose breath one shivered.

It was sunset when we passed the white town and whiter lighthouse of Messina. Over the dark outlines of the hills of Sicily the great golden orb sank into a sea of orange and pale lemon. On the right the Italian mountains glowed with rose and gold and amethyst. As the glow and glory faded and the boat headed into the grey of the open sea and gathering night, a chill not entirely of the evening stole over most of us. Home lay behind, the grey unknown before. Just then home and the homelike seemed best.

### Nursing and Companionship.

My first experience of nursing in Egypt was among friends in that part known as up the Nile, and, at first, I was required not so much for nursing as for companionship and care. It was still very hot—too hot to remove my charge to Cairo, so there on the outskirts of civilisation we remained for a time. Rising at 5 A.M. the day began with an hour's ride. At 6.30, after a cup of tea and bread and butter (prepared by ourselves), Mr. —— set off to superintend the work he had in hand. At 11 o'clock Mrs. —— and I lunched, after which we retired to bed till 3 o'clock, when I made afternoon tea. Till you have simmered at about 100° you have never duly prized tea. Then came two or three hours spent in the verandah whilst we worked or read. Dinner by moonlight at 8 P.M., when we were generally joined by some friends, usually ended the day.

Alexandria and Cairo I enjoyed, and I left kind friends there, but these are more European than Eastern, and I liked that first bit best. It is only when one gets away into the regions beyond, where one still finds old forgotten temples undiscovered yet by tourists, and the desert stretches from one's door, that one really finds Egypt, and even then

### NURSING PRIVATE PATIENTS IN EGYPT.—*Continued.*

it is modern, permeated with Western bustle and energy, its very climate, changed by irrigation, growing humid. Baby's arrival took place in Cairo. There, with every assistance, comfort, and convenience at hand, my part—about which I had been pretty nervous—was simple, and all went well. With baby's advent my work was at an end. Having no desire to remain in Egypt I did not seek employment, but, as among my friends and acquaintances were one or two medical men, I found my services more than once called into requisition.

### Difficulties of Language and Food.

My first case—a gentleman suffering with concussion—was staying at a good hotel in Cairo. Everything one could possibly need or wish for was obtainable for the asking. An eminent doctor was in charge of the case, so that, apart from the usual anxieties attendant on so critical a case, there were no especial difficulties to encounter. My next experience was very different—a Greek lady laid up with pleurisy. My patient, who was very ill, spoke little or no English. Her household—like most—consisted entirely of native servants. These, unlike ours, are non-resident, with the exception of the boy who, sleeping in a wooden erection outside, is supposed to keep guard over one's slumbers. They go home at night, and it used to seem to me half the day, for directly each one had performed his own especial duty he departed till such time as it again required doing. With Arabic I had made little progress, and I had to bethink me of all I was likely to require during the day and secure it before Mr. —— departed in the morning. A good nurse is supposed to anticipate her patient's needs, but it is not always possible, and to have to stand by helpless, unable to understand, vainly trying one thing after another, only to be met by a piteous shake of the head, is a dreadful experience, but out there a very common one. Nourishment was another difficulty. My patient's ideas and mine differed diametrically on this subject; but as the very foods, let alone their nourishing qualities, were unknown to me, it ended in her living principally on figs, eggs, white wine, sweet cakes, and Turkish coffee—a sweet sticky compound which, served in the tinest of cups, I found generally took the place of tea among my foreign friends. My own chief food was fruit, bread and tea, as I did not take kindly to Greek-Egyptian cookery. I was glad when the advent of a niece from Smyrna released me, for the position was unsatisfactory.

### The Next Case.

After this came a second-class hotel at Port Said, where every language under the sun save English seemed spoken. The case was a premature confinement complicated with enteric. Dust, discomfort, noise, mosquitos, and loneliness—for my patient, taken ill whilst travelling, was a stranger—formed but a short list of my troubles. Moreover, cow's milk was unobtainable, the ass on which for a time we relied seldom turning up at the proper moment. I have often wondered how my poor little patient ever did pull through it all; that she did so was as much due to the doctor's kindness as to his professional skill, to the comforts and dainties that were daily sent from his own house. The attack of enteric was only slight; but how the temperature dodged up and down, leaping from 99° to 105° seemingly without rhyme or reason. But a brave little soul, and with much to live for, she struggled gallantly and made a splendid recovery to my intense satisfaction; her case closely resembling another patient's where with premature confinement was severe malaria, this latter case of course not in Egypt.

### No Scarcity of Nurses.

With regard to nursing out there, English nurses are few and far between, but the supply seems so nearly to meet the demand, that I would advise no nurse to go out without a definite assurance of employment from more than one doctor. Where the household work is entirely done by men, ladies undertake duties they would not do at home, and in cases of illness, invariably when possible, come to one another's help. Above all, let no nurse go out without her L.O.S. certificate, and a wide and varied experience of maternity cases. Her work will consist mainly of such cases, and there—under new and unknown conditions—it is terribly easy for things to go wrong. Distances are great; medical aid seldom immediately obtainable. Symptoms which here might mean little or nothing being apt at times to indicate grave danger. Experience in fever nursing is also indispensable.

There is much too to contend with. The heat for eight months is trying even to English women who can take their ease. The language difficulty is a very real one, as I found to my cost; the life is apt to be lonely. Even when one has friends, they are away many months in the year; in the cool season they are full of engagements. In Cairo and Alexandria, there are small homes to which English nurses are attached, and one or two nurses find it pays to winter there, but it only just pays, for though fees range from three guineas to seven per week, expenses are high.

### A New Name.

By the way, I have been called by many names; but in Egypt I had a brand new one. I was a Hakinda. One never quite knows what thoughts lie behind these impassive Eastern faces, but my experience was that to be a Hakinda one was only a little below a Hakin, and was to be thought very highly of indeed, though it was a perpetual puzzle to the Oriental mind why the Hakinda would persist in tidying rooms and making beds and washing patients' cups and glasses herself instead of letting the housemaid do it; but then the Hakinda was only a woman after all.

### A Question of Clothing.

With respect to clothing, white dresses are by far the best—as colours fade very quickly—with grey alpaca for travelling. For winter, light tweed or serge is needed, and warm wraps are necessary; for underwear I found a mixture of thin silk and wool the most comfortable. I have not referred in any way to nurses connected with the civil or military or such native hospitals as the Kuser-el-Aini. These ladies are afforded ample opportunity for sharing in the general social life, and things are certainly made as pleasant for them as circumstances permit. Friends of mine, too, travelling with patients, have spent most enjoyable winters in Egypt.

### To Nurses.

We invite contributions from any of our readers, and shall be glad to pay for "Notes on News from the Nursing World," or for articles describing nursing experiences, or dealing with any nursing question from an original point of view. The minimum payment for contributions is 5s., but we welcome interesting contributions of a column, or a page, in length. It may be added that notices of appointments, entertainments, presentations, and deaths are not paid for, but that we are always glad to receive them. All rejected manuscripts are returned in due course, and all payments for manuscripts used are made as early as possible after the beginning of each quarter.

### A Visit to the Hospital for Incurables at Naples.

BY AN ENGLISH NURSE.

During a recent stay in Naples I visited the Incurable Hospital, and had the good fortune to be accompanied round the building by a member of the medical staff. The hospital was founded in 1521 by Francesca Maria Luogo, and was originally intended for incurable cases only, but it has been gradually transformed into a General Hospital, with additional wards for maternity cases. The building is large enough to contain 2,000 beds, but the average number occupied is about 1,200. Its situation is obscure, like many of the hospitals in Naples, and it would be quite overlooked by strangers unless they happened to hear of its existence. The hospital has a rich endowment, and is never in need of money; this extraordinary state of things is very prevalent in Italy, where they evidently manage these matters differently, whether to the advantage of the general public or not is of course open to question.

### Long and Lofty Wards.

The wards are simply splendid, the larger ones containing 82 beds, and the smaller ones 48. The floors are of marble and the walls are whitewashed. To the eye of an English nurse those long and lofty wards, with their marble floors and spacious width, present glorious opportunities for spotlessness and order, and visions of light-footed probationers neatly robed in becoming uniforms flitting about hither and thither came unbidden to my mind as I meditated on what "might have been." These were the kind of wards that would respond to a London Sister's touch. At the side of each bed is a slab of marble on a bracket which does duty for a locker—no clothing is kept in the wards—and in addition to the treatment cards and number over each patient's bed, is a large square slate upon which is inscribed the name and disease of the patient. About the middle of each ward a space of four yards square is curtained off, which serve as an operation room for minor cases, and here the larger and more important dressings are done.

### No Privacy.

It gave me a slight shock to see a doctor sitting on a patient's bed with his hat on, smoking a cigarette, while the "Soeur de Charité" prepared a wound for his inspection. Perhaps he believed in smoke as a disinfectant, and possibly he was only a student. Bedside screens are an unknown luxury in Italian hospitals, and so the patient knows no privacy and dies as he has lived, exposed to the gaze of his fellow creatures. I tried to recall what I had seen of the everyday life of the Italian poor, how they scorn the use of blinds, and eat, drink, work, and perform their toilet in full view of every passer-by, if not actually on the pavement, and thus consoled myself that they probably do not miss delicate attentions to which they have never been accustomed.

### No Flowers nor Plants.

In one small ward was a patient who had undergone ovariotomy. She looked happy and comfortable, and seemed to be doing well. It appeared to be the custom to isolate these cases for the first few days and then to remove them to the general surgical ward. In the male wards the small iron bedsteads are covered with coloured striped rugs, and in the female wards with white coverlets. No flowers or plants relieve the monotony of the long rows of beds, "whiteness, whiteness everywhere," absolutely no attempt at decoration of any kind beyond the inevitable altar-table with its gaudy tinsel and painted images of the Virgin Mary, etc.

### The Nurses.

Some of the patients looked very ill indeed but all seemed well cared for and contented. The nursing is in the hands of religious Sisters who assist the doctors with dressings, prepare bandages, keep instruments clean and polished and are responsible for medicines, temperatures, etc. They wear washing dresses, large white aprons and the usual voluminous caps with flapping wings. Upon inquiry I was told that they had certain hours off duty but the time must be spent in religious exercises. Morning prayers begin at 5.30 A.M. They get no holidays and are only allowed to see their friends in the presence of the Mother Superior. Their sleeping accommodation is in the adjoining convent. They receive no salary but, on the contrary, pay varying sums, in some cases as much as ten thousand francs (four hundred pounds) for the privilege of devoting their lives to the service of the Convent. Though their hours on duty are very long, they are not subject to so much fatigue as English nurses and have no responsibility with reference to training their subordinates. They are assisted by Camerieras who do all the menial work and are very poorly paid.

### A Pathetic Sight.

The Children's Ward is large and airy and occupied by about 25 children, who appeared for the most part to be suffering from some form of tuberculosis. This was perhaps the saddest sight of all in that great hospital, so many little victims of hereditary disease looking prematurely old and stunted in mind and body. The Sister-in-charge, a kind, motherly woman, seemed very fond of her little flock and was busy putting on nice holland pinafores and pretty pink and blue frocks. The little iron cots with their white coverings looked clean and tidy, and nearly all the patients were up and dressed. The sister told me that some of them had been there for years and would remain until they died many of them were incurable cases. The eldest girl acted as assistant nurse and was highly commended for her kindness to the little ones. One little laddie was pointed out to me as a specially interesting case. He was about ten years old and was suffering from "myxœdema"; the poor child could hardly walk, his legs were so abnormally large; in fact his entire person was very swollen, and his skin of a peculiar reddish-blue tint. Altogether he presented a very pitiful spectacle. The Sister said he was better than when he was admitted, and had lost several pounds in weight. I saw no toys, but hoped there were some behind the scenes; it was nearly dinner-time, so perhaps they were put away.

### Paying Wards.

The maternity block is quite shut off from the rest of the building. A small operation-room, containing a specially constructed table and very little else, a long narrow ward where all the patients sleep previous to delivery, a labour-room, and private rooms for the doctor and nurses complete this set of wards. The infants are all sent to the "Annunziata," a kind of foundling hospital in connection with the "Incurabile," where the mothers too are sheltered until situations are found for them. There are also a few paying wards which are occupied by private patients who pay from 7 to 10 francs a day. These patients have special privileges in the matter of visitors, and enjoy the luxury of a room to themselves, besides sundry extras in the way of diet. The heavier meals are served at 10 and at 3; coffee and bread at 8 A.M. and milk at 6 P.M. The kitchen is in charge of two Sisters, who are assisted by male cooks and kitchen boys. I saw large quantities of macaroni, without which no Neapolitan considers his diet complete. The bread for the day is distributed to each patient in the morning, and reposes somewhere or other within reach until disposed of.

### Nursing and Social Life.

BY A HOSPITAL NURSE.

ALMOST every earnest member of the nursing profession during her training is sooner or later confronted with this question: "Is it possible for her to continue her social life during her allotted times off duty?" It is a difficult question and one well worth consideration. In the first place let us consider the type of woman who would make the ideal nurse, in a few words the best, physically, mentally, and morally. It is commonly said that nursing demands all, not part. And yet, is this fair to the woman? She gives of her best, and in return what does she receive? At the end of her period one finds a skilled nurse, certainly, but in too many cases, physically and mentally a weaker woman than before. And why? Because that woman has been under the impression that she could not live two lives. Her times off duty she spends recruiting her physical self so as to be equal to the strain under which she is constantly labouring. As a natural result she becomes narrow-minded, for is she not living in a groove? She is voted uninteresting by those outside her profession—and with reason too—for has she not ceased to take an interest in all things apart from the little world in which she lives?

Now, on first consideration this seems certainly an evil—but a necessary one. How often is the probationer advised to put her heart and soul into the work? "You think too much of your times off duty," she is told. Nursing means sacrifice, it is said, and every nurse who has the training of probationers entrusted to her will probably aver that the best nurses are those who have a strong incentive and purpose in their work, those who have put, and do put, aside every other consideration for it. Also we blame those amongst us who do not take the work seriously enough, whom we find thinking of other things than nursing, and we conclude, neglect their work in consequence, which is perhaps in many cases more a natural conclusion than a perfectly correct statement. The pity of it is that this inevitable absorption comes gradually, and is discovered, alas! often too late for remedy. Now, how do outsiders regard this type of nurse? As a good woman, certainly—useful when people are ill, uninteresting, incapable of discussing subjects of general interest; in fact a nurse, and a nurse only.

Yet one is not to imagine for one moment that this woman is what she appears to be, but such is the impression she too often makes, because the isolated life she has led has made her lose confidence in herself except in her one life interest, and it is this want of confidence generally, and inability to interest herself in matters outside her own sphere that causes her to be misinterpreted.

Now, has this evil a remedy? I think it has. But our system of hospital training needs considerable reform before it can be remedied, and it rests with the authorities of the hospitals to help their nurses to become—not mere machines, but women, intelligent in their work. Let them fully realise their responsibilities in that direction, but at the same time, teach them to be fully alive to the progress in the world outside. Encourage them to read and think, form libraries for them, reading circles, give them opportunities to attend concerts, lectures, etc., give their higher natures a chance to develop. After all, the majority are young women with responsibilities much the same as their fellow-sisters. Their work has grand developments, and the more their sphere is enlarged the greater their possibilities. In this way, and this way only, can their social status be improved. As our present system stands little can be done. It is not longer hours off duty that are necessary, it is the encouragement to utilise those hours to the best advantage. The probationer should be taught to put her whole energies into her recreation as well as into her work. She must realise that though her work fills the greater part of her life, she has still other duties which she owes to herself and to her friends. Let her see that her work would gain and not lose by it, that the added variety in her life would make her fresher, more intelligent, more energetic, and of wider sympathies generally: these in addition to the physical benefits which she would derive from it.

When this has become the rule and not the exception, then, and then only, will nursing have reached its highest limits. In no other vocation has a woman greater opportunities of influencing those about her, and it is only the cultured woman—I use the word in its broadest sense—who is capable of using that influence to its best advantage.

As nursing now stands, many of us think with regret of our lives before we became nurses, when we used to read and think, when our little lives embraced a wider horizon. The time comes when we are compelled to stand aside for a while, and though we love our work, and know that we should speedily become as engrossed as before, we wonder if it were necessary to sacrifice all that we have done. We have drifted away from those that were once so much to us. And why have we done it? Where was the necessity? How can we answer these questions satisfactorily?

I can only warn the intending probationer to go into her new life with her eyes open. Let her realise that it rests with her to make or mar her life. Her work should be much to her, but not everything. Let her pursue her vocation quietly and zealously, with enthusiasm, yet without that touch of fanaticism which is so often a feature of it, and thus she will be tending to fulfil her highest development, and reach the purpose for which God has made her.

### Appointments.

[No charge is made for announcements under this head, and we are always glad to receive, and publish, appointments. But it is essential that in all cases the school of training should be given.]

ALTON WORKHOUSE INFIRMARY.—Miss Beatrice White has been appointed superintendent nurse. She was trained at Farnham Union Infirmary.

MERCERS' HOSPITAL, DUBLIN.—Miss Constance Fullager has been appointed matron and lady superintendent. She was trained at the Clinical Hospital, Manchester, and the Children's Hospital, Great Ormond Street, London. She has since done private nursing for three years, has served in South Africa for a year, and received the South African medal, and subsequent to her return has been matron of the Sherbourne Sanatorium.

SHEFFIELD WORKHOUSE INFIRMARY.—Miss Agnes A. F. Clarke and Miss Mary Wright have been appointed charge nurses. Miss Clarke was trained at the Stanley Hospital, Liverpool, and has since been sister in the same institution, charge nurse at St. Elizabeth Home, Glasgow, and charge nurse at the South-Eastern Hospital, London. Miss Wright was trained at Barnsley Union Infirmary, and has since been staff nurse in the same institution.

### The District Nurse and the "Pianner Lidy."

A TRUE STORY.

ALL about the docks she was called the "Pianner-lidy." A pale, delicate girl of twenty-five, she spent her days trudging wearily from one public-house to another, playing for an hour or more at each, to amuse the Jacks ashore. And though her clothes were barely kept together by darns and patches, and though her poor tired feet peeped out through her shoes, yet the sailors were wont to say, "it was plain seen she was above the likes o' them." And they showed her a rough respect, marking their appreciation of her music by beating time on the floor with their heavy feet, and tapping their glasses on the bar for applause when she stopped. Many of them would lay a little coin, as if by accident, on the piano, which the "lidy" would pocket surreptitiously; and this was the way she earned her bare existence.

Now she was lying dangerously ill in her tiny garret, where the district nurse had found her. The first visit had proved that she was above the average of Nurse Wilson's usual patients, and by degrees her pitiful little story leaked out. The orphan daughter of a poor clergyman, she had become, at nineteen, companion to a rich old lady. Her life was that of a poorly paid dependant until, two years later, a nephew of the old dame's, himself a man of property, had loved her and asked her to be his wife. His aunt had accepted the inevitable with apparently good grace; but when, a few months later, her nephew was called away, she proved her falsity by upbraiding the girl most cruelly, and turning her out into the street. Friendless, and without a character, the orphan sought for work in vain. Too proud to apply to her *fiancé*, and half believing the assertion that such a marriage would "ruin his career," she lost herself in the vast city, shrinking from all intercourse with her equals, and finally drifting into the life of a street musician.

To-night she lay inert and still, unobservant of Nurse Wilson's movements, though now and again her eyes would open wide and stare with feverish eagerness.

"Nurse," she whispered at last, "am I dying?"

"You are very very ill, my dear," answered the nurse tenderly. "Is there anything you wish to tell me?"

"Yes," sighed the girl; "in that little wooden box—see, in the far corner—are the only treasures I could save. I want you to send them to *him* when I am dead, and say I loved him and was true to him always."

Then—her mind eased by a solemn promise—"the Pianner Lidy" turned to the wall with a smile on her white lips, and sank into a sleep of exhaustion.

At night in her own room Nurse Wilson sat by the fire, thinking deeply. At last she penned a letter.

"DEAR MR. KEITH, [it ran]—Amongst the poorest and most destitute of my patients in the docks is a girl named Ellice Wynne. She is in a very critical condition, and has asked me to send her few remaining treasures to you after her death. Thinking you may prefer to fetch them, I write to let you know.

"Yours truly,

"DISTRICT NURSE WILSON."

The nurse's eyes were sad, but she smiled as she wrote. And the man came.

. . . . . . . . .

Up and down the broad terrace they pace slowly, her arm in his—a bride and groom of one spring. It is a beautiful June evening, when the whole world seems made for lovers, and all around lies the hush of a perfect bliss. The sun woos with delicate glow the plain beneath, and touches into radiance the horse-chestnut, as it stiffly waves to the blushing copper beech. The sturdy oak gazes in lordly pride at the shimmering raiment and supple grace of the Forest Queen, while on the hill beyond, the stern dark pines stand in protecting nearness to the delicate larches. In the garden diminutive lobelias glance perkily up at some haughty geraniums, and a few tall jonquils bend their heads to look into the blue eyes of the forget-me-nots. The tulips stand stately and alone—the bachelors and spinsters in a Garden of Love—but as the young couple stroll happily by, the roses nod their heads with glee, and the lilies exchange a knowing glance, while the discreet pansies turn their wise faces away.

And as the lovers pause to hear the passionate song of a blackbird, he draws his young wife closer, and exclaims half in fear: "To think I might never have found you!"

"Dear!" she whispers in reply, "we owe it all to Nurse!"

### Everybody's Opinion.

**[Correspondence on all subjects is invited, but we cannot in any way be responsible for the opinions expressed by our correspondents. No communication can be entertained if the name and address of the correspondent are not given as a guarantee of good faith, but not necessarily for publication. All correspondents should write on one side of the paper only.]**

### THE HOME SISTER'S AUTHORITY.

"NURSE ELIZABETH" writes: I was much interested in a letter in last week's HOSPITAL *re* "Home Sister's Authority." The position of home sister has always seemed to me to be one most difficult to fill. By many she is looked upon as housekeeper, someone to complain to about the food, clothes lost in the laundry, etc., it being seemingly forgotten that her position is one of the highest in the hospital. As to the complaint of "A Liverpool Nurse," I am of opinion that the home sister was but fulfilling her duty in speaking as she did. I am sorry any nurse should have to be spoken to about wearing a sailor hat on Sunday. If nurses wear private dress they should consider wet Sundays when choosing their clothes, and eschew flowers and feathers. It should be remembered by nurses that when they sign an agreement to work for an hospital they are under the control of their superiors, both on and off duty, until that time has expired; also that "training" does not only mean nursing, but tidiness, punctuality, etc., and they should endeavour to heighten the standard of the hospital they serve by their behavious, dress, etc., off duty as well as on. Dirty boots speak for themselves. Nurses are very ready to carry their complaints and grievances to the home sister, but they resent any compluint she may make to them. I am sure that many home sisters will agree with me that their position is not easy and requires all their tact, patience, and endurance

### NURSING HOMES IN TOWNS.

"OBSERVER" writes: In a philanthropic age I am struck by the fact that people with the most excellent intentions often act very selfishly even when they are impelled by an unavowedly altruistic spirit. My observation is at present focussed on the thoughtless manner in which houses are taken as a home, or homes, for incurable and sometimes dying people close to other houses in a road or terrace. There appears no trouble in carrying out this arrangement if the landlord is willing, but I cannot help thinking that other healthy tenants of neighbouring houses should have some voice in the matter. They have practically none now. Often a house long unlet and out of repair is taken for this purpose. Take for instance the many homes which are opened all over London and the provinces for children suffering from tuberculosis in various forms. An ordinary house with ordinary sanitary arrangements is taken—its normal household would be perhaps at most ten persons; this number when the home is opened is frequently doubled and before long trebled. A second house is taken next door after a short time with equally insufficient sanitary arrangements. It does not seem to occur to the good people who are taking these houses how much better and fairer to the community it would be to wait a little till they find sufficient money to take a house which stands by itself, preferably in the country. Children would recover much better in pure good air; if incurable, they would benefit from a good garden, and the rents asked would be much more moderate. Having had experience of work in a small incurable home in a London square I can honestly say I pitied the patients—and our neighbours. There were in this home thirty-six cases, mostly tubercular, with open sores requiring great attention. We had cases of hip disease, curvature of spine, phthisis, and many bedridden paralytic patients. The drainage was quite suitable for a family of the ordinary size, but not for us, and I cannot think that either the cases or our unfortunate neighbours benefited by our presence. I am glad to say that this home has now been moved to the country. I wish many would follow the example and do away with what is a distinct danger. No house should be allowed to take in tubercular or other cases without inspection, which should I think be repeated annually, so that if necessary some addition could be made to the ordinary sanitary arrangement of the house. In any case, space, sunshine, light, and air are all good gifts for the sick, and these we can get far more cheaply and abundantly in the country than in any large town. "To hasten slowly" would be a wiser policy.

### NURSING AMONG THE POOR.

"A PUPIL MIDWIFE" writes: I should like to say that the suggestion that "L.O.S." makes to nurses who are going in for midwifery training in the London slums is an exceedingly good one, for if they could only realise one quarter of the misery and poverty they were going to encounter in their work they would thankfully accept the smallest sums from friends to be used for their patients. Many and many a time I have found a shilling or two spent on mother lying there helpless in the midst of her poverty and squalor, has brought comparative happiness and comfort, and has considerably assisted in a speedier recovery. No one can imagine, who has not been doing the same sort of work, how terribly a midwifery nurse is handicapped if she has no money to enable her to occasionally buy bare necessities. Nurses with private incomes, however small, are certainly much to be envied, and even then it seems very terrible how little one can do to alleviate the misery and poverty all around you. Only too often it is the breadwinner for the family, of very often eight or nine, who is your patient, and not only is there nothing in the way of food for the patient but nothing for the family as well. It is very seldom that a family can afford to occupy more than one room. The rents in my particular district are so enormously high, that you can see almost at a glance how much, or rather how little, there is in the cupboard. I have known a family of seven inhabiting one small room certainly not nine feet square—the only window in it being one pane of glass stuffed up with rags—and paying 4s. 6d. a week for it. I have often explained to my patients how much cheaper it would be for them to live a little farther out of London, and how much better it would be for the children, but the same reply is always given: first the husband's train or 'bus fare would cost too much to make it any cheaper, and then, when I have proved that such would not be the case, they say, "Well, you see, nurse, we do like society, and it would be so dull in the country," and nothing will alter their idea. But apart from this, their pluck and enduring powers are sometimes marvellous, as I think the following incident will show. One November evening I was summoned to a woman living in Drury Lane. On arriving at the house I had some difficulty in finding my patient's room, everywhere being in darkness. At last I happened to knock at the right door, but on opening it, to my surprise, found there was still no light in the room. I began to think there must be a mistake, but a voice said, "Is that you, nurse, for if it is, will you please stop there a minute until my neighbour comes back? She has just gone to fetch a light, as my lamp has just fallen down from the ceiling and exploded; and please nurse, the baby is born, and I am lying under the table." All this was said in the calmest way imaginable, as though either incident were quite an every-day occurrence. When at last the light arrived I did, indeed, find the poor creature lying half-way under the table, with all her outdoor clothes on, and the baby as nearly smothered as it was possible for it to be, considering that no one had dared to touch it. While taking my patient's clothes off, I asked her how it was she had not been able to manage better and to get at least some of her things off. So she said, "Well, you see, it was like this. My husband's out of work. We've got nine children, and this last week or two I have had good work given me cleaning gentlemen's offices in the City, so, of course, I wanted to keep on as long as ever I could. So I have been cleaning there ever since nine o'clock this morning until seven to-night, and directly I got inside the door I was took bad and couldn't get any further. I've been feeling a bit bad all day, but, there, I'd got a new pair of boots on, and I thought it must be them as made me uncomfortable." Not one word of complaint did she make, and before I left her was as cheerful and contented as possible. Two days after this my nurse, who had been with me at the time and was visiting the patient each day, came to me with a long face, and said she had just been to see this same woman, and found her sitting by the fire, fully dressed, and working for the children. I immediately put on my bonnet and cloak, and went round to see her, fully prepared to scold her, but when I got there I found her crying bitterly in bed, eight hungry children sitting round an almost empty table, and the husband—quite a young and decent sort of man—looking about as miserable as a human being could. So under those circumstances what could I say? I was obliged to explain to her that she had broken the rules under which I was working, therefore I should not be allowed to visit her again, but I think no one with a heart at all could help sympathising with such a mother when she assured me there was no one to do a thing for the children but herself. Soon after this incident I was nursing a private case not a mile away from this spot, and happened accidentally to hear the housekeeper say one morning that the vegetable for one course at dinner the night before had cost £15. The contrast between the two cases seemed too terrible for words, and how true it is that "How little one half of the world knows how the other half lives."

### Presentations.

ON the resignation of Miss Birdsall Hobkinson from the post of lady superintendent at the Ipswich Nurses' Home, to become lady superintendent of the Staffordshire Institution for Nurses, Stoke-on-Trent, the nursing staff presented her with a handsome silver kettle and stand, much regretting her leaving, and wishing her every success and happiness in her new appointment.

### Wants and Workers.

CAN anyone give, or sell cheaply, a small wicker bath-chair for little boy of five years' old, suffering from tuberculosis? If so, please write to Nurse Laurence, Melrose, Whyteleafe, S. O., Surrey.

### Echoes from the Outside World.

### The King's Cruise.

THE King on his Scotch cruise last week reached Ballachulish, the *Victoria and Albert* being anchored in Loch Leven on the morning of Saturday, His Majesty proceeding in a pinnace to Mamore Deer Forest, 12 miles up the Loch, on a shooting expedition, but unfortunately the wind was contrary and there was no sport. He returned to the yacht in the afternoon, the Queen going to meet him and acknowledging the greetings of the numerous people who had assembled in the hopes of seeing her, by waving the bouquet which she carried in her hand. On Sunday morning the Royal yacht left her moorings, and proceeding down Loch Linnhe and through the Sound of Mull, round Ardnamurchan Point, so passed out between the islands of Cauna and Runo. Anchor was afterwards struck for the night in Uig Bay, in the north of Skye. The inhabitants did not at first realise that the King was among them, and when his Majesty landed the only person in sight was the local policeman, with whom the King entered into conversation. Later in the evening the inhabitants showed their loyalty by lighting a bonfire in honour of the occasion. On Tuesday morning Stornoway was reached, and the Provost, two bailies, and the town clerk came on board, and the King announced his intention of landing in the afternoon and driving through the town to Lewis Castle. The inhabitants gave their Sovereign a most enthusiastic reception.

### The King's Bible.

THE Bible to be presented to the King by the British and Foreign Bible Society as a memento of the Coronation will be on view at the Bible House, 146 Queen Victoria Street, E.C., from September 22 to September 27. The volume has been bound in the finest pressed royal-red morocco, with bevelled boards, gilt edges, richly inlaid, and hand-tooled throughout. The design is adapted from early Christian symbols and ornamental work to be found in the catacombs and buildings of the first to the fifth centuries. The central device is a cross of early shape, enriched with an interlaced pattern in gold, on grey-blue inlay. Around the cross are vine leaves and bunches of grapes, emblems of Christ, the True Vine. Surrounding and framing the above is a border composed of wheat ears, typical of the Bread of Life, upon which are ten emblematic medallions. The book has been bound under the personal direction of the Society's president, the Marquis of Northampton.

### Armenian Gift to the Queen.

THE Queen has accepted from Armenian women at Constantinople employed by the "Friends of Armenia" Association, a Coronation gift which they desired to send her in order to show their gratitude to England for the help given them since they lost their relatives in the massacre. It is in the form of a beautiful cushion embroidered with the Royal Arms, and Miss Charlotte Knollys, writing to Lady Frederick Cavendish on behalf of her Majesty, asks Lady Frederick to assure the poor Armenian women at Constantinople that the Queen will consider it "as one of her most valued gifts."

### Aftermath of the Coronation.

THE KING has presented Coronation medals to the men and women servants of the Royal Household at Windsor Castle and elsewhere. It will be learnt with interest that the statues representing the seven Edwards which were in the entrance to the annexe to Westminster Abbey, erected for the Coronation, have been removed to Windsor Castle. The annexe itself has been sold for old builder's materials, the only portion unsold being the flagstaff, for which an insufficient figure was bid.

### The National Memorial to Queen Victoria.

THE love and admiration which still gather around the name of Queen Victoria is taking very tangible shape in the colonies. Canada has already promised a contribution of £30,000 towards the National Memorial in Commemoration of Queen Victoria to be erected in the capital of the Empire, whilst Cape Colony will contribute £20,000, New Zealand no less than £15,000, and Natal £10,000. As yet the amount to be contributed by Australia has not yet been fixed, but it will probably be on the same liberal scale, and already the sum given by the self-governing colonies exceeds £100,000.

### Movements of Royalty.

LAST week Princess Henry of Battenberg, in the capacity of Governor of the Isle of Wight, dedicated to the public a recreation ground, eight acres in extent, presented by Mr Tankerville Chamberlayne, and laid out by the Newport Corporation at a cost of £4,000. The Princess having alighted from her carriage, unlocked the gate of the ground, and said, "I declare this ground open for the public in accordance with the terms of the gift, and I also declare, in accordance with the approval of the King, that it shall be called the Victoria Recreation Ground." In the evening there was a carnival, procession, and military tattoo.

### Mr. Chamberlain and the St. John Ambulance Brigade.

ON Saturday afternoon Mr. Chamberlain presented war medals to 50 members of the Birmingham Corps of the St. John Ambulance. After the ceremony, which took place at Highbury, Mr. Chamberlain delivered a short address, in the course of which he referred to the excellent work done by "that great and charitable order, St. John of Jerusalem." He went on to say that it would have been a great satisfaction to him, on the occasion of his recent accident, if he had found anyone near to him to give him the aid which the members of the order are so well qualified to afford. Having alluded to the "unexampled and unparalleled calls" which the war had made upon the national resources in all directions, he continued, "In my judgment public opinion in this country never has, and never will, submit to the expenditure which would be necessary if we were always fully to be prepared for such an exceptional emergency as that which we have just come through, and accordingly it will always be necessary for us in similar circumstances to do what we did then, and to call upon the voluntary patriotism of a free people to supplement the necessary deficiencies in the regular service." That call, the Colonial Secretary declared, was the one which the St. John Ambulance Brigade was summoned to meet, and he dwelt with satisfaction on the fact that something like 2,000 members of the organisation went to the front to render assistance, and throughout the heat of the strife carried on the pure work of humanity, and gave their help indiscriminately and impartially to friends and foes alike.

### Death of a Famous Animal Painter.

THE death took place on Sunday of Mr. J. T. Nettleship, the popular animal painter. Mr. Nettleship, who was one of four brothers who all attained distinction—the only survivor being the eminent occulist—commenced his career as a literary man, and his "Essays on Browning" was almost the first attempt at a detailed study of the poet. But he soon relinquished it, and took to painting. Trained at Heatherley's and the Slade school he soon became known as an exhibitor at the Grosvenor, the Royal Academy, and the New Gallery. His special study was wild animals, and his beasts of prey—notably lions, polar bears, and leopards—always attracted attention and commanded a measure of admiration. He certainly had learnt to understand animals, and his vigour of imagination was indisputable.

### For Reading to the Sick.

"CONSIDER THE LILIES OF THE FIELD."

FLOWERS preach to us if we will hear:—
The rose saith in the dewy morn:
I am most fair;
Yet all my loveliness is born
Upon a thorn.
The poppy saith amid the corn:
Let but my scarlet head appear
And I am held in scorn;
Yet juice of subtle virtue lies
Within my cup of curious dyes.
The lilies say: Behold how we
Preach without words of purity.
The violets whisper from the shade
Which their own leaves have made:
Men scent our fragrance on the air,
Yet take no heed
Of humble lessons we would read.

But not alone the fairest flowers:
The merest grass
Along the roadside where we pass,
Lichen and moss and sturdy weed,
Tell of His love who sends the dew,
The rain and sunshine too,
To nourish one small seed.

I can never remember the time when I did not love her, this mother of mine with her wonderful garments and ordered loveliness, her tender care and patient bearing of man's burden. In the earliest days of my lonely childhood I used to lie chin on hand amid the milkmaids, red sorrel, and heavy spear-grass listening to her many voices, and above all to the voice of the little brook which ran through the meadows where I used to play: I think it has run through my whole life also, to lose itself at last, not in the great sea but in the river that maketh glad the City of God. I feel not so much desire for the beauty to come, as a great longing to open my eyes a little wider during the time which remains to me in this beautiful world of God's making, where each moment tells its own tale of active, progressive life in which there is no undoing. It is a time of exceeding peace. There is a place waiting for me under the firs in the quiet churchyard; thanks to my poverty I have no worldly anxieties or personal dispositions, and I am rich in friends. . . . I am most gladly in debt to all the world; and to Earth, my mother, for her great beauty. As I write the sun is setting; in the pale radiance of the sky, above his glory, there dawns the evening star; and the earth like a tired child turns her face to the beauty of the night.—*M. Fairless*

"LORD, I WILL FOLLOW THEE."

JESUS! my breath is failing; lead me on
Softly and gently, as my strength can bear;
Draw me to Thee in closer union,
And for eternal life Thy child prepare.
Let Thy love shine upon my soul, and chase
This mistiness and darkness quite away;
Till Faith discerns her holy resting-place
Distinctly, in the perfect light of day.
Robe me in snowy raiment; store my heart
With precious jewels from Thy treasury;
This world is not my rest, let me depart,
And let my ransom'd soul return to Thee.
Well may I trust Thee, Who Thyself hast given,
To gain for me the peace and bliss of Heaven.

*From the "Dove on the Cross."*

### Notes and Queries.

**The Editor is always willing to answer in this column, without any fee, all reasonable questions, as soon as possible.**

**But the following rules must be carefully observed:—**

1. **Every communication must be accompanied by the name and address of the writer.**
2. **The question must always bear upon nursing, directly or indirectly.**

**If an answer is required by letter a fee of half-a-crown must be enclosed with the note containing the inquiry, and we cannot undertake to forward letters addressed to correspondents making inquiries. It is therefore requested that our readers will not enclose either a stamp or a stamped envelope.**

*Hospital Training.*

(131) Will you tell me if a three years' certificate from a large infirmary, such as St. Pancras, would be recognised as full nursing qualification for a good post in a general hospital?—*A. G.*

Certainly.

I am anxious to enter a London hospital as probationer. Will you kindly tell me what influence is required and how could I get it?—*E. E. P.*

There is no influence required but merit. Apply to the matrons ot suitable hospitals, stating full particulars, and you will receive instructions as to what you must do. See "The Nursing Profession: How and Where to Train" for list of institutions, training nurses, conditions and terms.

I am 25 years of age, and have been engaged all my life in looking after children and in nursing adults in their own homes; I wish to take up nursing, medical gymnastics, or special kinds of cookery for the sick, and I have only £5 for the purpose. Will you kindly tell me what I had better do?—*X. Y. Z.*

There is no doubt that you had better enter a good training school as soon as possible. See reply above to *E. E. P.*

*Poor Law Publications.*

(132) 1. Will you kindly tell me if there is a Scotch or Irish paper like the English "Poor Law Officers' Journal," as I am desirous of securing a post under either one or the other, and I would be glad to know where I can find out about them. 2. I signed as cottage nurse for three years at 8s. a week. The Association promised to find me work, and it was arranged that if I left before my time was up I was to forfeit £5. I have to pay my own board, which is 6s. a week. Since last August I have been at home for 11 weeks, and have had to pay £2 14s. for board. Can I leave the Association now without paying the £5?—*E. W.*

1. Messrs. King, Orchard House, 2 and 4 Great Smith Street, Westminster, will be able to give you information about Poor Law publications. 2. The facts stated seem to point to an injustice, and we think that there must be some mistake. If the Association promised to keep you in work and have not done so, then they have broken the contract; it is, however, a matter on which only one conversant with all the facts could advise.

*Male Nurse.*

(133) Will you kindly tell me of a hospital in London or elsewhere where male nurses are trained, and where a Medico-Psychological certificate can be gained?—*B. G. F.*

The National Hospital for the Paralysed and Epileptic, Queen's Square, Bloomsbury, W.C., is the only hospital for training male nurses. Some of the larger asylums (for lists see "The Nursing Profession: How and Where to Train") prepare attendants for the certificate of the Medico-Psychological Society.

*Up-country Nursing Association.*

(134) Will you kindly give me the address of the Up-country Nursing Association?—*India.*

Apply to Mrs. Sheppard, 10 Chester Place, Regent's Park, N.W.

**Standard Nursing Manuals.**

"The Nursing Profession: How and Where to Train." 2s. net; post free 2s. 4d.
"A Handbook for Nurses." (Illustrated). 5s.
"Nursing: Its Theory and Practice." (New Edition.) 3s. 6d.
"Nursing in Diseases of Throat, Nose, and Ear." 2s. 6d.
"Surgical Ward Work and Nursing." (Revised Edition.) 3s. 6d. net; post free, 3s. 10d.
"Art of Massage." (Second Edition.) 6s.
"Elementary Physiology for Nurses." 2s.
"Elementary Anatomy and Surgery for Nurses." 2s. 6d.
"Practical Handbook of Midwifery." 6s.
"Mental Nursing." 1s.
"Art of Feeding the Invalid." 1s. 6d.
All these are published by the SCIENTIFIC PRESS, Ltd., and may be obtained through any bookseller or direct from the publisher 28 and 29 Southampton Street, London, W.C.

### Travel Notes.

**By Our Travelling Correspondent.**

### CVIII.—AMONG THE HIGHLANDS.

You can see all the places of which I am about to speak with ease in three weeks, and the cost need not exceed £16 including your ticket, which will be within a few shillings more or less of £3. The temperance hotels are the most moderate, and with management you can live at them for 8s. per day. In the large cities like Edinburgh and Glasgow you can find accommodation considerably cheaper, because there is competition, but not so in provincial districts. I will give you an outline of my last tour, which was arranged on these lines as to expense. It is a sad pity that Scotland is such a dear country in which to travel, for there is, I really believe, none more beautiful, but prices are almost prohibitive for slender purses; not only are hotels dear, but carriages, steamers, etc. . . . . all partake of the same disease.

### A Few Hours in Perth.

Before proceeding to any of the charming resting places along Tay-side time your arrival in Perth so that you may have a few hours to see the different places of interest before taking the Highland railway to Pitlochrie, etc. . . . I take for granted (although in these degenerate days I am not sure of being correct in that certainty) that you are all readers of the "Waverley Novels," and admirers of "The Fair Maid of Perth" will follow up with interest the various spots connected with her history. Curfew Street still exists, and her house is pointed out. The fine old church of St. John, not then built, stands on the site where the gallant Smith passed from the High Street to Curfew Street to greet his love on St. Valentine's day. Arrived there, he found the Duke of Rothesay climbing the window of Catherine's room, assisted by Sir John Ramorny whose hand the redoubted Smith then and there hewed off. The mutilated ruffian was hurried off by his friends in iniquity, to the Blackfriars monastery, which was not many years later the scene of the murder of James I., the second son of the mild but incompetent Robert III., whose eldest son, the Duke of Rothesay, who figures in Scott's novel, fell a victim to political intrigue and family greed, and perished in the most terrible manner of starvation in the Palace of Falkland. Scone, some two miles from Perth, is hardly worth visiting; a perfectly modern and uninteresting castle has replaced the old abbey, and we have the stone of tradition on which our king was lately crowned in Westminster Abbey. The following singular prophecy relates to this stone—

> Except old seers do feign,
> And wizard wits be blind,
> The Scots in place must reign
> Where they this stone shall find.

It is verified inasmuch as our Royal family are descended from James VI. of Scotland.

The Inches of Perth are two vast meadows immediately outside the city, and on the North Inch was fought that sanguinary battle lasting an entire day between the rival clans Chattan and Quhele in the autumn of 1396. It was considered, in the savage counsels of that age, that it was a good thing that these two powerful clans should fight till they were annihilated, and this actually happened, for of Clan Quhele but one man remained alive.

Of this fearful encounter, the masterly account in Scott's novel is doubtless drawn from historical sources. Peculiarly dramatic is the episode of the death of the rival Pipers. Considered to be too far advanced in years for actual combat, they remained at the edge of the enclosed space and cheered their clans by wildly impassioned music, but their shrill blasts inflamed their ready passions; they gradually drew nearer and nearer to each other, rage and fury burning in their aged eyes, flew at each other's throats, and in the struggle drew too near to the river, where they were afterwards found, fast locked in a deadly grip in the waters of the Tay. Sir Walter says the chanter of the Chattan pipes used on that occasion is, or was, in the possession of the chief of the Clan MacPherson—Chattan being the Gaelic for MacPherson.

### A Choice of Spots on the Highland Line.

At Pitlochrie go to Doggart's temperance hotel, where terms are fairly moderate, and enjoy the endless charming excursions to be made from there. The walk or drive to the loch and falls of Tummel is ideally beautiful, and it is the best spot for visiting the Pass of Killiecrankie, where the gallant but reckless Claverhouse fell to rise no more.

This walk is perhaps more enthralling than any other in the district on account of its historical associations. Walk from Pitlochrie to Blair Athol, which will take you right through the Pass, distance about seven miles. If this is more than you can manage, drive the first three and send the carriage on to meet you at the north end of the Glen. I walked all the way, and found it not at all fatiguing, and one can return entirely by rail.

There is a station at Killiecrankie, but if you go to that you miss the most interesting part, namely, the exact site of the battle, which raged about Urrard House—still a fine old house, though much altered since 1689. Not many years ago, during some alterations, two skeletons were found in a narrow secret passage, sword in hand; it was supposed that they had attacked each other there, and, both falling, their bodies had mouldered to decay, unknown to all.

The traditional spot where Bonnie Dundee met his death is a slight mound, with beautiful trees; he was killed by a bullet formed of a silver button, in accordance with the old belief of the Covenanters that his friend and protector, the foul fiend, rendered him impervious to any missile but one of the precious metal. Claverhouse fell in the moment of victory—a glorious death for a soldier—he was carried to Blair Castle, where he died a few hours later, and was buried in the old church.

Instead of Pitlochrie, you may like to make your headquarters at Boat of Garten, a small place on the moors farther north. It is more bracing than Pitlochrie, and therefore better liked by some, but the immediate surroundings are not so romantic, being far wilder, more bare, and of a sterner character altogether.

### TRAVEL NOTES AND QUERIES.

Is the Continent Cheap for Residence (Paardeberg)?—This is a question continually asked, and the answer is, it depends entirely on *how* you live. The great saving is, that there is not the same attempt to keep up appearances as at home. To begin with, people who keep three servants, or two and a boy, would only keep one; then you need not entertain, or your hospitality can be kept to afternoon tea. Rates and taxes are fewer, or at any rate smaller, and if you live in apartments, you almost entirely escape them. Education, and good at the price, is far cheaper. But now for the other side. Groceries are dearer, many items notably so; tea very dear and indifferent, biscuits the same, candles ditto. Fish and meat about the same as in England, poultry rather cheaper, milk and butter quite as dear, unless you are in a very primitive district. Concerning house rent, the advantage is *generally* slightly on the continental side, and markedly so if you go far afield and not near a large centre.

## Figures and Tables

**Fig. 58. f1:**